# Home, but Homebound: Identifying and Supporting Homebound Older Adults

**DOI:** 10.1093/geroni/igaf122.1027

**Published:** 2025-12-31

**Authors:** Jennifer Reckrey, Christine Ritchie

## Abstract

The homebound, defined as those who never or rarely leave their homes, represent a large and growing subgroup of older adults with complex care needs. As compared to those who leave home routinely, the homebound experience high chronic disease burden, significant functional impairment, and limited access to primary care and needed social services. In this symposium, we present innovative research about how to effectively identify and support this unique population. First, McManus et al. present a scoping review that reveals persistent care gaps among the homebound, including limited access to dental care, neuropsychiatric care, healthy foods, and social and functional supports. Second, Davoudi et al. describe risk factors predicting homeboundedness identified via machine learning models within the National Health and Aging Trends Study (NHATS), including physical performance, driving, ability to walk 3 blocks, functional dependency, and having low income. Next, we present three studies that also leverage NHATS to identify subgroups within the homebound with unique care needs and experiences. Leff et al. examine heterogeneity among the homebound by examining those who are not frail. Dr. Katherine Ornstein et al characterize the nearly 2.5 million decedents who report being bedbound in the last year of life. Finally, Dr. Junxin Li et al describe associations between sleep complaints and being homebound among older adults with multimorbidity. Taken together, these findings highlight the unique care needs of the homebound and underscore the need for targeting clinical innovations and policies to improve access to needed care and supports at home.

